# Regulation of in vivo dynein force production by CDK5 and 14-3-3ε and KIAA0528

**DOI:** 10.1038/s41467-018-08110-z

**Published:** 2019-01-16

**Authors:** Dail E. Chapman, Babu J. N. Reddy, Bunchhin Huy, Matthew J. Bovyn, Stephen John S. Cruz, Zahraa M. Al-Shammari, Han Han, Wenqi Wang, Deanna S. Smith, Steven P. Gross

**Affiliations:** 1 Developmental and Cell Biology and Physics, University of California, Irvine, CA USA; 2 Biological Sciences, University of South Carolina, Columbia, SC USA

## Abstract

Single-molecule cytoplasmic dynein function is well understood, but there are major gaps in mechanistic understanding of cellular dynein regulation. We reported a mode of dynein regulation, force adaptation, where lipid droplets adapt to opposition to motion by increasing the duration and magnitude of force production, and found LIS1 and NudEL to be essential. Adaptation reflects increasing NudEL-LIS1 utilization; here, we hypothesize that such increasing utilization reflects CDK5-mediated NudEL phosphorylation, which increases the dynein-NudEL interaction, and makes force adaptation possible. We report that CDK5, 14-3-3ε, and CDK5 cofactor KIAA0528 together promote NudEL phosphorylation and are essential for force adaptation. By studying the process in COS-1 cells lacking Tau, we avoid confounding neuronal effects of CDK5 on microtubules. Finally, we extend this in vivo regulatory pathway to lysosomes and mitochondria. Ultimately, we show that dynein force adaptation can control the severity of lysosomal tug-of-wars among other intracellular transport functions involving high force.

## Abstract (web summary)

Dynein plays roles in vesicular, organelle, chromosomal and nuclear transport but so far it is unclear how dynein activity in cells is regulated. Here authors study several dynein cofactors and their role in force adaptation of dynein during lipid droplet, lysosomal, and mitochondrial transport.

## Introduction

Cytoplasmic dynein (dynein-1, MAP1C) is essential for intracellular transport of organelles and other cargos toward the cell’s nucleus^1,2^. Together with the opposite directed plus-end kinesin family of motors, these molecules move along cytoplasmic microtubule (MT) highways, allowing appropriate cargo positioning and delivery.

Dynein plays roles in vesicular, viral, chromosomal, and nuclear transport and is essential for neuronal migration during cerebral development^3,4^. Owing to this diversity of roles, it is highly regulated, frequently via regulatory cofactors, including dynactin, LIS1, NudE (*NDE1*), and NudEL (*NDEL1*)^4^. Dynein forms two major complexes: with dynactin or with NudEL-LIS1^5–8^. Dynactin is important for increased processivity, and dynein–dynactin–BicD complexes can travel longer distances^9^. In contrast, the NudEL–LIS1 complex is important for force production. Deletions in the *LIS1* gene cause Miller–Dieker syndrome^10^, presumably because LIS1 enhances additivity of single-motor forces^11^, and thus facilitates dynein’s high-load function, which is important for the nuclear migration^2,12^ underlying neuronal migration; NudEL tethers LIS1 to dynein and helps regulate the dynein–LIS1 interaction. It is unclear how these two complexes (dynein–dynactin or dynein–NudEL–LIS1) coordinately regulate dynein. They may or may not function simultaneously: they share an either/or interaction site on the dynein intermediate chain (DIC)^6^, but LIS1 associates with moving dynein–dynactin–BicD2 complexes^13^. One model is that the two complexes multiplex or trade off binding with dynein, but how this might be regulated is not understood.

In addition to the dynein–NudEL–LIS1 core complex, there are other NudEL-interacting proteins that provide further regulation^14,15^. In neurons, the signaling kinase cyclin-dependent kinase 5 (CDK5) phosphorylates NudEL^16,17^. However, the mechanistic implications of this phosphorylation are controversial with respect to the effect on MT-dependent cargo transport in axons. Klinman et al. suggest that NudEL phosphorylation by CDK5 increases dynein–NudEL–LIS1 affinity and locks dynein in a nucleotide-bound state that decreases processive motion of various dynein cargos^17^. In contrast, Pandey et al. suggest that CDK5 phosphorylation of NudEL leads to increased dynein activity by promoting a high-affinity dynein–NudEL–LIS1 complex, which then increases transport by dynein^16^. Most recently, CDK5 phosphorylation of NudEL was found to be critical for rerouting mis-sorted dendritic cargo out of the axon initial segment (AIS), a dynein-dependent process^18^. However, mechanistic interpretation of observed neuronal effects due to altered CDK5 function is difficult, because in addition to any potential NudEL phosphorylating role, CDK5 phosphorylates Tau, causing its release from MTs (and hence promotes subsequent MT depolymerization)^19–23^. Any role for CDK5-mediated control of dynein in non-neuronal cells is unknown. Because the main activators for CDK5—P35 and P39—are only present in neurons, it has been assumed^24^ that CDK5 may not be important in non-neuronal cells. However, evidence for pleiotropic non-neuronal roles for CDK5^25^ supports a re-evaluation of this assumption.

Assuming a phospho-regulated dynein–NudEL–LIS1 complex, said complex could be further modified by 14-3-3ε^15^, since clinically, many dynein-related neuronal diseases are made worse by 14-3-3ε impairment. For instance, decreased 14-3-3ε protein levels result in a worsened lissencephaly phenotype in LIS1-deficient patients^26^. Further, 14-3-3ε mRNA expression levels are decreased in the prefrontal cortex of schizophrenic and bipolar patients^27,28^. Lewy bodies, abnormal protein aggregates found in Parkinson’s disease nerve cells, contain 14-3-3ε^29^. Thus 14-3-3ε is an actively studied target in neurodegenerative and neuropsychiatric diseases. However, little is known about its function in intracellular transport. Interestingly, 14-3-3ε interacts strongly with phospho-NudEL to promote normal dynein complex localization and activity^14^. Further, NudEL can be dephosphorylated by the phosphatase PP2A, and 14-3-3ε protects phospho-NudEL by sterically inhibiting PP2A’s access to the phosphorylation sites^26^. In addition to providing insight into the identified pathway for force regulation in cells, these studies have mechanistic implications in multiple diseases, where transport is likely to be important. CDK5 is implicated in diabetes^25,30–33^, neurodegenerative diseases^34^, and cancer^35^, and 14-3-3ε is an important risk factor in schizophrenia^27^.

All of this leads to the model—relevant in both non-neuronal and neuronal cells—tested in this work. Our hypothesis is that the recently described force adaptation of cellular lipid droplets (LDs)^11^ occurs because CDK5 becomes activated, phophorylates NudEL, thus increasing the affinity of NudEL for DIC, and through increased NudEL–DIC interactions, promotes dynein’s utilization of the NudEL–LIS1 system. Further, we hypothesize that NudEL’s phosphorylation is protected by 14-3-3ε. We use LDs in COS-1 cells as a model system, because their motion and protein makeup is well understood, their motion plays a known role in metabolism^36^, they are amenable to force measurements in cells^11^, and there is no Tau in COS-1 cells. Therefore, alteration of CDK5 signaling does not alter the MT cytoskeleton, in contrast to what occurs in neurons^37^. While studied in LDs, we further hypothesize that this regulatory pathway is generally important for other dynein cargos and thus test it in the context of lysosomes and mitochondria. We find that CDK5, 14-3-3ε, and KIAA0528 are all essential for dynein force adaptation and that they regulate the transport of LDs, lysosomes, and mitochondria.

## Results

### Force adaptation in wild-type COS-1 cells

**Fig. 1:**
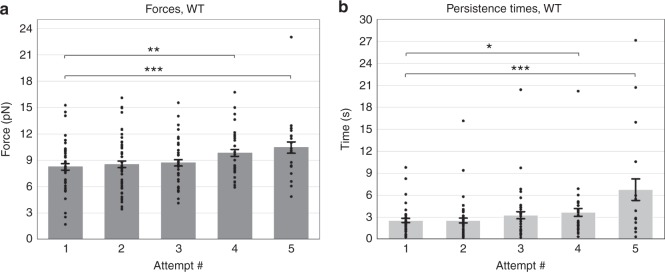
Minus-end forces and persistence times increase when dynein-driven LD cargo are under load. Average peak forces for minus-end moving LDs show slight increase in WT background (**a**). Average persistence times for dynein-driven LDs show significant increase in WT background (**b**). Quantified data represent the mean ± s.e.m. of *n* = 5 independent experiments. *T* test *P* values *P < 0.05, **P < 0.01, ***P < 0.001

We previously showed that, under load, both forces and persistence times (the time over which the force is maintained) in dynein-driven LD cargos increase at each attempt^11^; the increase is typically first apparent in attempt 2 or 3 and easily statistically significant by attempt 4 or 5 (Fig. 1, Supplementary Figure 8). This trend occurs when measurements are made both in unperturbed wild-type cells and in control cells treated with transfection reagent (Supplementary Figure 1).

### CDK5 is essential for dynein force adaptation

**Fig. 2:**
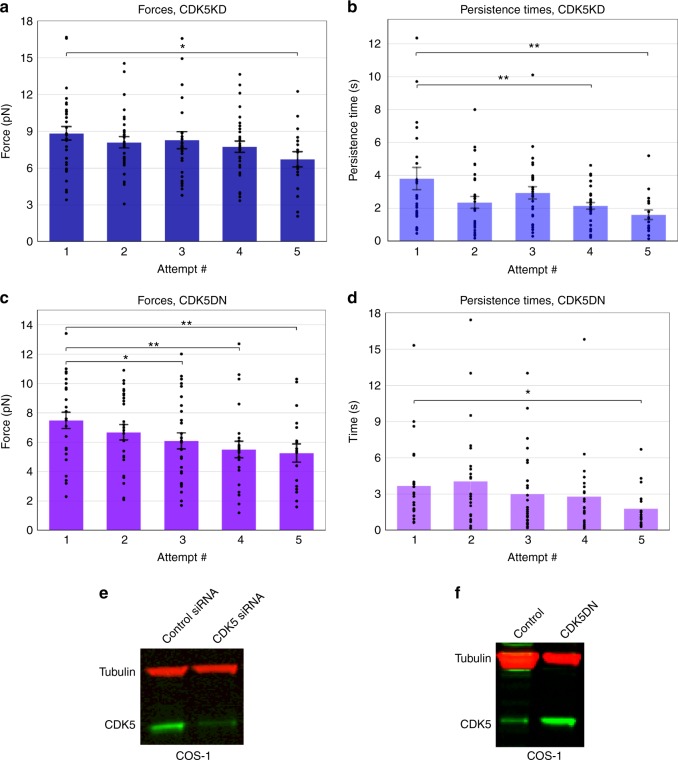
CDK5 is essential for dynein force adaptation in COS-1 cells. Average peak forces for minus-end moving LDs decrease at successive attempts in CDK5 knockdown background (**a**) and CDK5dn overexpression background (**c**). Average persistence times for dynein-driven LDs show a significant decrease in CDK5 knockdown background (**b**) and CDK5dn overexpression background (**d**). CDK5 knockdown was >90% effective (**e**), and CDK5dn overexpression was successful (over 15× increase in the expression of control) (**f**). Quantified data represent the mean ± s.e.m. of *n* = 5 independent experiments. Two-sided *t* test *P* values *P < 0.05, **P < 0.01

**Fig. 3:**
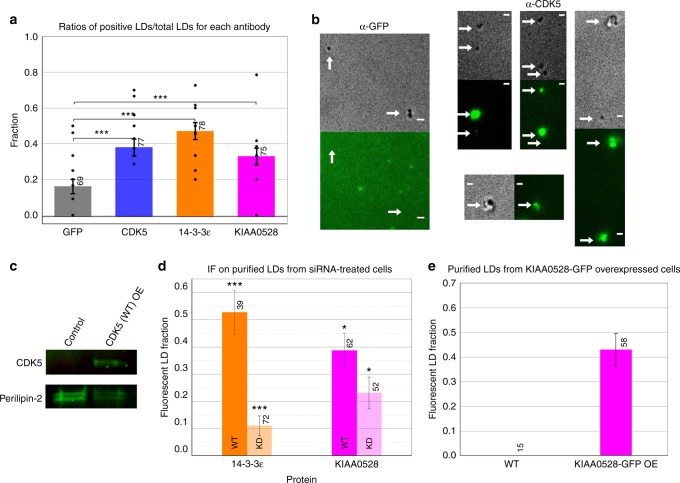
CDK5 and 14-3-3ε and KIAA0528 are found on lipid droplet (LD) cargo. Intact purified LDs were probed with antibodies against CDK5, 14-3-3ε, KIAA0528, and GFP (negative control), the percentage of LDs that had detectable immunofluorescence signal quantification is shown in **a** and sample immunofluorescence images are shown in **b** with 2 μm scale bars, and white arrows designate lipid droplets. To assess CDK5 localization to LDs, proteins were precipitated from purified LDs and analyzed by WB. Perilipin-2: (an LD marker) is present on both samples and 25× CDK5 is present on the LDs from the CDK5 overexpression background (**c**). To assess 14-3-3ε and KIAA0528 localization, IF experiments were performed on LDs purified from wild-type or siRNA-treated cells. LDs from the KD cells showed reduced levels of the respective proteins, quantified in **d**, with example images for each experiment in the supplement, Fig. 6. To further confirm KIAA0528 LD localization, LDs were purified from wild-type (WT) or KIAA0528-GFP overexpressing cells, and the percentage of LDs with GFP puncta was quantified. LDs from KIAA0528-GFP cells show increased puncta compared to WT cells (**e**). Representative fluorescence images are in the supplement, Fig. 6. Quantified data represent the mean ± s.e.m. of *n* = 2 independent experiments. Two-sided test of proportions *P* values *P < 0.05, ***P < 0.001, ****P < 0.0001

CDK5 phosphorylation of NudEL has been shown by three separate groups^24,38–40^. Thus we explored the possibility that CDK5 phosphorylation of NudEL is important for dynein force adaptation. We knocked down endogenous CDK5, and under such conditions, we found that dynein force production does not increase at each attempt as for the wild type but instead forces and persistence times decreased with subsequent attempts (Fig. 2a, b, Supplementary Figure 5, Supplementary Figure 8). The same phenotype was observed when we blocked CDK5 activity by overexpressing a previously characterized dominant-negative CDK5 construct (CDK5dn)^16^ (Fig. 2c, d, f, Supplementary Figure 8). Neither CDK5 knockdown (KD) nor CDK5dn overexpression altered plus-end forces (Supplementary Figure 2). Thus inhibiting CDK5’s kinase activity coincides with alteration of dynein force adaptation in cells, where forces and force durations decrease rather than increase. After conducting immunolocalization on purified LDs, we found that CDK5 was indeed present on the LDs, supporting this CDK5 regulatory function (Fig. 3a–c, Supplementary Figure 6). We confirmed CDK5’s presence on LDs by purifying LDs from control cells and cells overexpressing a wild-type CDK5 construct and conducting a western blot (WB) on the precipitated protein from these LD purifications. We found perilipin-2 in these blots, confirming that we had indeed successfully purified LDs. The CDK5 signal is enriched in the LDs from the CDK5 overexpression sample (Fig. 3a).

### 14-3-3ε is essential for dynein force adaptation

**Fig. 4:**
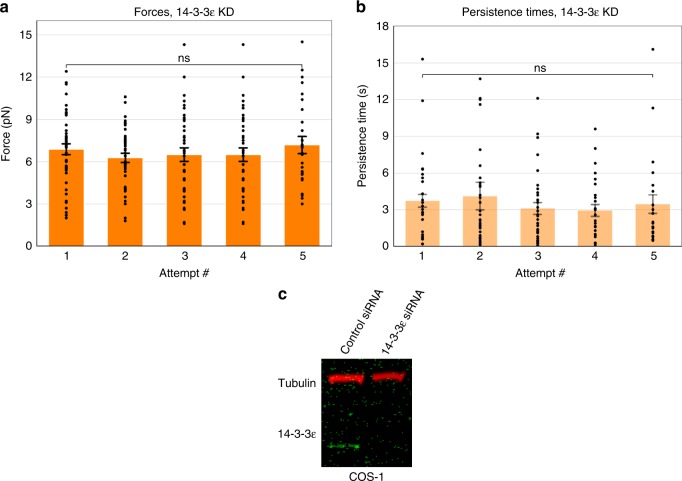
14-3-3ε is essential for dynein force adaptation in COS-1 cells. Average minus-end peak forces (**a**) and persistence times (**b**) at each attempt do not increase in the 14-3-3ε knockdown background. 14-3-3ε knockdown was >80% effective (**c**). Quantified data represent the mean ± s.e.m. of *n* = 4 independent experiments. All two-sided *t* test *P* values >0.05

We next examined 14-3-3ε, since in a neuronal context, it was previously reported to protect phospho-NudEL^15^. Consistent with a potential role in LD function, we found that 14-3-3ε was present on purified LDs (Fig. 3a-c, Supplementary Figure 6); cellular KD of 14-3-3ε led to its disappearance on purified LDs, as detected by immunofluorescence. When we knocked down 14-3-3ε (Fig. 4), the result was reminiscent of that observed for the CDK5 KD but less severe: in contrast to wild-type behavior, neither forces nor persistence times increased at successive attempts (Fig. 4a, b, Supplementary Figure 8), though they did not decrease as was the case for CDK5 KD. Again, no obvious effect was observed on plus-end force production (Supplementary Figure 2).

### CDK5 cofactor KIAA0528 is necessary for force adaptation

**Fig. 5:**
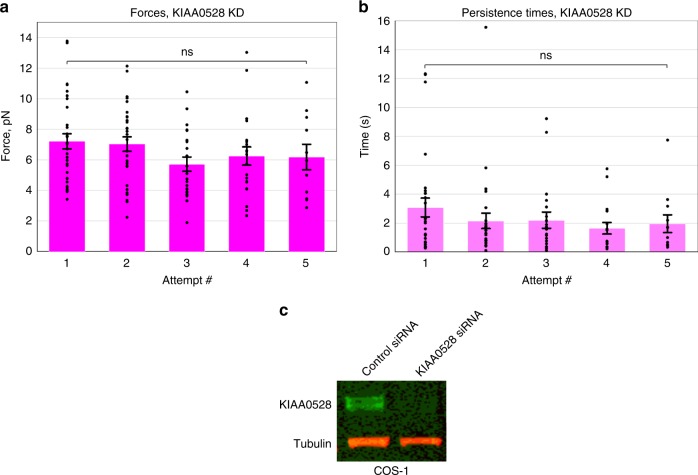
KIAA0528 is essential for dynein force adaptation in COS-1 cells. Neither average minus-end peak forces (**a**) nor persistence times (**b**) at each attempt increase in the KIAA0528 knockdown background. KIAA0528 knockdown was >99% effective (**c**). Quantified data represent the mean ± s.e.m. of *n* = 4 independent experiments. All two-sided *t* test *P* values >0.05

Since CDK5 activity is highly characterized in neuronal cell types but less is known about its function in non-neuronal cells, we became interested in factors contributing to its non-neuronal activity. Additionally, as CDK5 is a known key regulator for the dynein motor^16–18^, further characterization of CDK5 could lead to novel dynein-regulatory mechanisms. We searched the CDK5 interactome via recent proteomic analysis of CDK5-associated protein complexes and prioritized prey candidates that are known to have MT-related cellular functions. This led us to KIAA0528 for multiple reasons: (1) it was identified as the top binding protein^41^, (2) this interaction was validated with anti-KIAA and anti-CDK5 immunoprecipitations (IPs)^41^, and (3) KIAA0528 is involved in GLUT4 transport^42^, a motor-dependent process relevant in diabetes. KIAA0528 is also potentially involved in cancer metastasis as it plays roles in cell proliferation and migration^43^. However, as a studied protein, its function is relatively unknown. As KIAA0528’s interaction with CDK5 had been confirmed via multiple approaches^41^, it seemed likely that if present, it might alter CDK5 function. We found KIAA0528 on purified LDs in COS-1 cells (Fig. 3a, d, e, Supplementary Figure 6), positioning it to contribute to LD transport. We then knocked down KIAA0528 (Fig. 5c) and conducted force measurement experiments in this background. The effect of loss of KIAA0528 was similar to that of 14-3-3ε: adaptation was eliminated, and neither forces nor persistence times changed (Fig. 5, Supplementary Figure 8). As for the CDK5 and 14-3-3ε KD backgrounds, the perturbation of KIAA0528 function did not alter plus-end force production (Supplementary Figure 2).

### Functional importance of NudEL phosphorylation

**Fig. 6:**
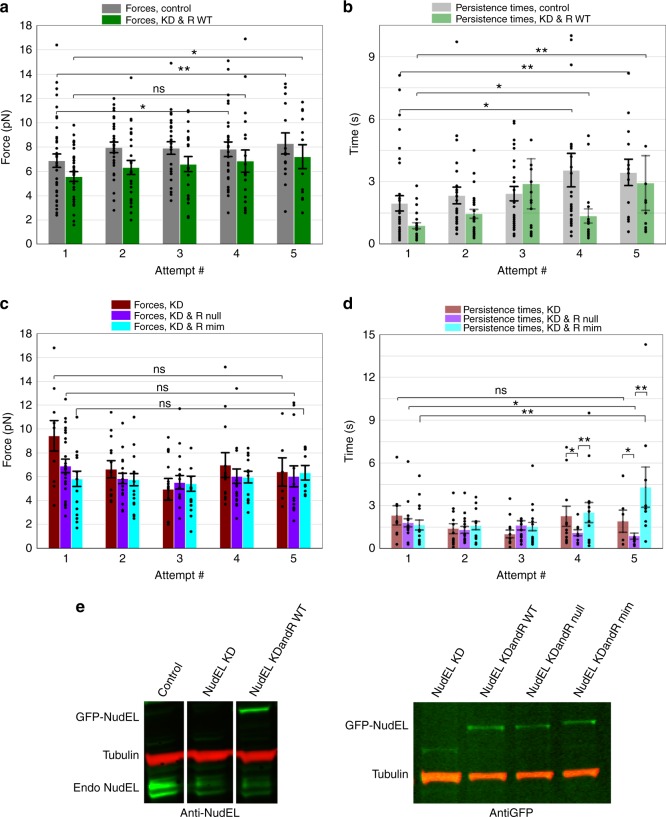
NudEL knockdown and replacement force profiles depends on NudEL phospho-state. Average minus-end peak forces (**a**) and persistence times (**b**) for control and NudEL knockdown and NudEL-WT replacement backgrounds. Average minus-end peak forces (**c**) and persistence times (**d**) for NudEL knockdown and NudEL knockdown and phospho-null and phospho-mimetic replacement backgrounds. Average endogenous NudEL/NudE (Endo NudEL/NudE) knockdown was 70%; average exogenous replacement (GFP-NudEL, band seen in NudEL KDandR WT lane) was 102% wild-type NudEL levels (left). WB probed for GFP found replacement with WT, null, and mimetic mutants resulted in similar expression levels (right). **e** Quantified data represent the mean ± s.e.m. of *n* ≥ 3 independent experiments. Two-sided *t* test *P* values *P < 0.05, **P < 0.01

NudEL and its sister protein NudE contain 55% identical protein sequence, but recent knockout (KO) mice of the respective proteins have suggested that the two might have slightly different functions^44,45^. Specifically, NudE KO mice are viable but have smaller brains than control mice^44^; however, NudEL KO mice are embryonic lethal^45^. If dynein is unable to adapt in the specific KD of NudEL, perhaps this suggests that force adaptation is in fact an essential dynein function. We previously reported^11^ that dual KD of NudE and NudEL eliminated force adaptation. Here we find that knocking down NudEL alone also abolished force adaptation (KD in Fig. 6c, d, Supplementary Figure 8). We suspect that our NudEL KD was specific to NudEL as none of the four NudEL small interfering RNA (siRNA) target sequences used were found in the NudE mRNA sequence. Thus dynein force adaptation specifically requires NudEL. The above data show that CDK5, 14-3-3ε, and KIAA0528 are all also vital for dynein force adaptation. Because CDK5 and 14-3-3ε are reported to affect NudEL phosphorylation in a neuronal context, and KIAA0528 is a CDK5-interacting protein potentially contributing to CDK5-mediated phosphorylation, we hypothesized that the overall impairment in force adaptation in all of these backgrounds reflects a loss of CDK5-mediated NudEL phosphorylation. Because the CDK5 phospho-sites on NudEL had previously been determined^24^ (and confirmed in multiple other publications^38–40^) to directly assess the ramifications of such phosphorylation events, we carried out force measurement experiments after knocking down endogenous NudEL and replacing it with either NudEL-GFP-wild-type (KDandR-WT), NudEL-GFP-phospho-null (KDandR-null), or NudEL-GFP-phospho-mimetic (KDandR-mim) variants. Because NudEL dosage matters, the replacement constructs were expressed at levels comparable to endogenous NudEL expression (Fig. 6e), and all mutants were expressed at similar levels to each other.

Importantly, under KDandR-WT conditions, we found that both forces and persistence times increased at successive attempts, similar to the control condition (Fig. 6a, b, Supplementary Figure 8). Since force adaptation was lost due to the NudEL KD (KD in Fig. 6c, d, Supplementary Figure 8), this result showed that our rescue construct was indeed functional. Under KDandR-null conditions, force adaptation was impaired: forces did not increase and persistence times decreased significantly (KDandR-null in Fig. 6c, d, Supplementary Figure 8). However, under KDandR-mim conditions, we partly rescued force adaptation: forces did not significantly increase, but persistence times increased by attempts 4 and 5 (KDandR-mim in Fig. 6c, d, Supplementary Figure 8). Importantly, our data indicate that the phospho-null rescue construct is functional: replacing with the phospho-null mutant decreases persistence times relative to the KD alone. Further, combined, the results confirmed the importance of the phospho-sites but modify our initial model that CDK5-mediated phosphorylation alone accounted for force adaptation (see Discussion).

### The CDK5 pathway is important for other dynein cargo

**Fig. 7:**
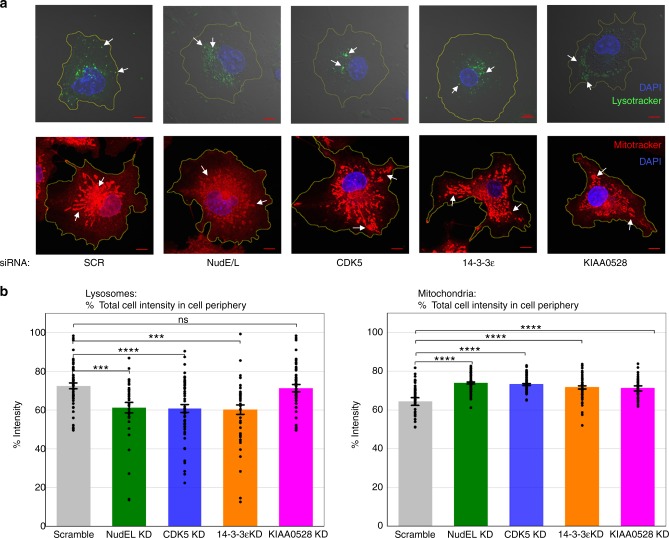
CDK5-activated dynein affects lysosome and mitochondria distribution in live cells. Representative images of cells in various backgrounds stained with DAPI and either LysoTracker-Yellow or MitoTracker-Red with 10 μm scale bars and white arrows to point out example lysosomes and mitochondria (**a**) and quantification of lysosome or mitochondria distribution (**b**). Quantified data represent the mean ± s.e.m. of *n* = 2 independent experiments. Test of proportions *P* values ***P < 0.001, ****P < 0.0001

Having extensively characterized the role of CDK5, 14-3-3ε, and KIAA0528 in dynein force adaptation with LD cargos, we wondered whether this regulatory pathway was important for other dynein cargos. We thus examined lysosomes and mitochondria. First, we conducted KD experiments and visualized the cargos’ spatial distribution in each case. Loss of NudEL affected the distribution of both cargos (Fig. 7, Supplementary Figure 7), confirming that NudEL contributes to their positioning. Further, loss of CDK5 activity altered the distribution of both lysosomes and mitochondria in a manner quantitatively similar to the effects of loss of NudEL for both classes of cargos. Loss of 14-3-3ε also had a comparable effect to NudEL and CDK5 KDs (Fig. 7, Supplementary Figure 7). The lysosomal redistribution may reflect a feedback effect: in the wild type, plus-end and minus-end runs are slightly longer than in the CDK5 KD background (Supplementary Figure 3c, d), possibly contributing to increased lysosomal dispersion. While we do not fully understand why there is a difference in the lysosome and mitochondrial distributions upon force adaptation impairment, the fact that there is a significant difference suggests that, overall, NudEL utilization is being regulated by CDK5 and 14-3-3ε and that this, among other things, contributes to positioning of multiple cargos. Intriguingly, there was a differential effect due to the loss of KIAA0528: its loss had no effect on lysosomes but altered mitochondrial distributions similarly to CDK5 and 14-3-3ε loss. Thus we concluded that, as for the LDs, KIAA0528 is required for NudEL–CDK5–14-3-3ε utilization in the mitochondria, but the lysosomal NudEL–CDK5–14-3-3ε pathway functions without it.

### The NudEL/CDK5 pathway contributes to lysosomal tug-of-war

**Fig. 8:**
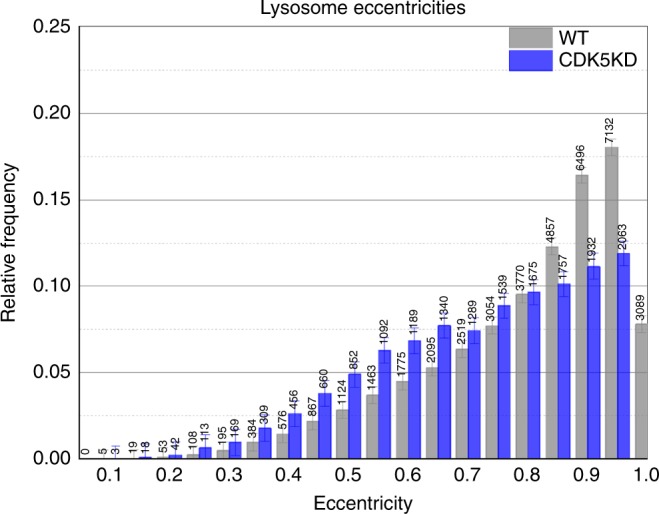
Lysosome deformation is reduced in CDK5 knockdown background. Histogram of lysosome eccentricities in control cells (gray) and CDK5 knockdown cells (blue). Eccentricity of 0 is a perfect circle, and eccentricity of 1 reflects highly elongated lysosomes; note the increased frequency of large eccentricities (gray bars at 0.9, 0.95, 1) in the control relative to the CDK5 knockdown. Distributions were determined to be very different (KS test, *P* < 0.0001) Quantified data represent *n* = 2 independent experiments

There is an ongoing controversy in the field about the extent to which opposite-polarity motors engage in a tug-of-war, and if so, the importance of such events, and how such tug-of-war events are regulated. We had previously published that such tug-of-war events do not appear important for LD motion^46^; but others reported that tug-of-war events do occur for endosome and phagosomes^47,48^. Since lysosomes are similar to endosomes and phagosomes, we reasoned that perhaps tug-of-war events between kinesin and dynein occur here too. Because the NudEL–CDK5–14-3-3ε pathway improves dynein’s force production, one might expect that the tug-of-war magnitude would be decreased when dynein’s force production is impaired. If one assumes that the plus-end and minus-end motors are randomly placed on the cargos, tug-of-war should lead to cargo deformations. Thus, to test this possibility, we developed a quantitative approach to measure lysosomal deformation in the time-lapse images by using custom written MATLAB code for image processing (see Methods). We found that we could indeed detect numerous elongated lysosomes and that the extent of such deformations was decreased in the CDK5 KD background, consistent with a decrease in the magnitude of the tug-of-war events (Fig. 8, Supplementary Figure 4). Thus the data suggest that tug-of-war does occur between motors on the lysosomes and that, by tuning dynein’s function, the CDK5 pathway can tune the severity of the tug-of-war. In principle, this change in apparent stretching could be simply due to decreased dynein utilization; however; this appears unlikely because initial forces (reflecting the number of engaged motors before adaptation) were not significantly decreased in LDs from control vs CDK5 KD LDs (compare attempt 1 forces in Figs. 1a and 2a, Supplementary Figure 2), particle tracking found no difference in the overall number of moving lysosomes nor in the pause durations (Supplementary Figure 3a, b). Run lengths do appear slightly shorter in the CDK5 KD (Supplementary Figure 3c, d) in both travel directions. Interestingly, although decreased CDK5 activity diminishes the magnitude of tug-of-war events (as judged by the decreased magnitude of deformations), this tug-of-war property change did not appear to alter directional switching probabilities (Supplementary Figure 3e), perhaps suggesting that the termination of a tug-of-war is regulated by a switching complex rather than load-induced stochastic disengagement of the dynein or kinesin motors. In conclusion, our data suggest that tug-of-war occurs for dynein-driven lysosomes, that the magnitude of severity of the tug-of-wars can be controlled via the CDK5 pathway, and that effect of such tug-of-wars on the properties of overall lysosomal motion is quite subtle.

## Discussion

Here we investigated the regulation of dynein force production for LDs, mitochondria, and lysosomes; it is partly controlled by CDK5/14-3-3ε, with KIAA0528 also contributing to the first two. Overall, our data are consistent with a model where CDK5, 14-3-3ε, and KIAA0528 are constitutively present on the cargo (Fig. 3, Supplementary Figure 6). Dynein force adaptation occurs when the presence of an obstacle induces CDK5 activation, and thus NudEL phosphorylation (which increases the affinity of NudEL for DIC^40^). This increased interaction facilitates a dynein-NudEL-LIS1 complex, with subsequent improved dynein force production (as previously described in vitro^2^). Our work shows the importance of NudEL phosphorylation and the proteins involved—CDK5, 14-3-3ε, and KIAA0528: without these proteins, dynein can no longer increase its duration of force production nor adapt its maximal forces. Consistent with this, alteration of the NudEL CDK5 phosphorylation sites alters the magnitude and duration of ensemble dynein force production (Fig. 6).

Our data suggest a two-step process for dynein force adaptation. First, our data shows that force adaptation requires increased utilization of NudEL/LIS1; for such utilization to occur, NudEL must be phosphorylated by CDK5, with 14-3-3ε, and KIAA0528 contributing to control of this pathway. Second, before engaging NudEL, dynein is likely working with a different cofactor (likely dynactin); we believe that, for successful force adaptation, Cofactor 1 must be released, in a CDK5-independent manner. Such a hypothesis is consistent with all of our data to date. First, we note that, in the CDK5 KD or CDK5dn overexpression backgrounds, force production becomes increasingly impaired with successive attempts (Fig. 2). This appears consistent with increasing release of Cofactor 1, without the ability to concurrently bind NudEL (due to its lack of phosphorylation); since we have blocked CDK5 activity in these backgrounds, the Cofactor 1 release does not depend on CDK5 activity. Such a model is also consistent with the NudEL phospho-null and phospho-mimetic force measurements: we see increasing deterioration of duration of force production (in the phospho-null case) and increasingly improved duration in the phospho-mimetic case, consistent with increasing release of Cofactor 1, paired with inability/ability to utilize NudEL phospho-null/phospho-mimetic.

Our hypothesis about modulation of Cofactor 1’s involvement obviously raises questions about the role of dynactin in the force adaptation process. Dynactin (with BicD) is reported to improve dynein force production in vitro and in vivo^49^. If dynactin were playing a significant role here, one might have expected a significant drop in overall force production in the P150 KD background, which was not observed in our past study^11^. Instead, forces and persistence times adapted similarly to the wild type^11^ (Fig. 3a, c). Overall, if there were simple competition between binding NudEL and dynactin, controlled predominantly by the DIC–dynactin interaction, we would have expected altered (perhaps improved) adaptation in the P150 KD background, which did not occur. Thus we favor the hypothesis that dynactin is not contributing significantly to dynein force production and that adaptation requires both release of dynactin and increasing utilization of NudEL controlled by phosphorylation. We note that our published data suggest that dynactin is somehow involved in adaptation: while its decrease did not alter the magnitude or duration of force production, it did alter the time between force production events^11^ (Fig. 2h). Certainly, future work will need to investigate the control of dynactin utilization, and with this tool in hand, it may then be possible to unambiguously disentangle the coordinated sequence of events proposed above.

Having determined that CDK5 does control force production for LDs, we then established that it was a generalized pathway contributing to positioning control of other dynein cargos (Fig. 7). We analyzed its contribution to lysosomal motion in more detail and found that lysosomal motors appear to engage in a tug-of-war and that, by altering dynein force production, CDK5 can tune the severity of such tug-of-war events (Fig. 8).

Our findings have multiple implications. First, by directly measuring dynein cellular force production and showing that CDK5 controls it in non-neuronal cells, we establish the CDK5 pathway as a validated mechano-control signaling pathway. This is important because previous results in a neuronal context had been contradictory, with some suggestion of CDK5 improving function^16^ while other work suggested CDK5 activation impaired dynein function^17^. We suggest that these disparate neuronal results may reflect combined effects of the increase in dynein function we observe, coupled with confounding effects due to Tau-detachment-mediated disintegration of the MT cytoskeleton. Certainly, in non-neuronal cells where we sidestep such cytoskeletal disintegration, our data suggest that CDK5 activation can locally increase ensemble dynein force production—one signature of which, for LDs, is force adaptation.

Second, assuming that these roles are indeed carried over into neurons, our data provide mechanistic insight into neuronal function and how alteration of these specific proteins could lead to neuronal impairment. For instance, 14-3-3ε is a schizophrenia risk factor, and our data suggest that its loss will prevent dynein force production upregulation; thus, when evaluating candidate processes potentially altered by 14-3-3ε decrease, one may want to focus on those requiring high forces, such as large cargo transport, MT reorientation, etc. Similarly, CDK5 was recently shown to play an important role in proper MT orientation in the AIS^18^. This role for CDK5 in facilitating high-force events may help explain why this process requires CDK5, as changing MT orientation likely necessitates high dynein force production.

Third, the importance of upregulating dynein force production may vary locally. Cells typically have regional variations in the densities of organelles and cytoskeletal structures. For example, in COS-1 cells, the perinuclear region has higher densities than the cell periphery. Assuming cargos can engage multiple MTs simultaneously, these differences in cytoskeletal architecture may mean that force adaptation becomes important as dynein cargos approach the nucleus. In axons, dynein and its cargo may encounter blocks such as protein aggregates or stalled organelles, and there are regions that appear to have higher organelle and cytoskeletal density, including axonal branch points and AISs. In AISs, actin patches are thought to halt vesicles carrying dendritic proteins^50^, which may involve force adaptation allowing dynein to overpower kinesin plus-end motors in these patches. Local initiation of dynein force adaptation as motors encounter high-density cytoplasm may thus be a critical role for the CDK5/14-3-3ε pathway.

Fourth, our data provide an intriguing framework for interpreting a previous report, which found that increasing neuronal CDK5 activity (via expression of its activator, P25) increased the number of stalled lysosomes^17^. Above, we suggested that this effect could result from CDK5-mediated phosphorylation of Tau causing Tau release and subsequent depolymerization of MTs. With decreased tracks, transport would decrease. However, alternatively, since we found that lysosomal-bound motors engage in moderate tug-of-war under normal conditions and that the severity of those tug-of-war events is controlled by CDK5 activity, it seems possible that tuning CDK5 activity could control dynein properties to avoid severe tug-of-war. This allows lysosomes to avoid concomitant dramatic stalling, which has been reported to occur in unregulated competition between kinesin and yeast dynein^51^ (working with NudEL and LIS1, cytoplasmic dynein functions more like yeast dynein (in the sense that it has a slow detachment under load)). Thus a second interpretation of the above-mentioned neuronal experiments suggests that increased CDK5 activity results in slower dynein detachment under load and even more severe tug-of-war between lysosomal motors, resulting in increased numbers of stalled lysosomes. Further, this neuronal observation that increasing CDK5 activity leads to stalls—which could result from unresolved tug-of-war—provides an intriguing model for why control of CDK5 activity is so important, why its alteration may easily lead to problems, and why local force adaptation may be needed. If CDK5 is aberrantly active, dynein is too powerful, and competitions between dynein and kinesin cannot be well controlled. Thus we hypothesize that dynein-mediated transport is tuned—via CDK5 activity/14-3-3ε levels and localization—to provide a moderately robust transport system with the ability to overcome typical obstacles to motion. We hypothesize that the role of force adaptation, then, is to prevent dynein from being too strong, so that tug-of-war typically resolves without aggravated traffic jams; it is only after repeated stalls (indicating a significant barrier) that maximum adaptation engages, subsequently allowing for increased dynein performance.

Fifth, for bi-directionally moving cargos, there is typically tight coupling between plus-end and minus-end motor activity:^52,53^ when one upregulates or downregulates one set of motors, there is usually feedback (though unknown mechanisms) resulting in concomitant changes in the opposite direction. Interestingly, CDK5 modulation of dynein force production appears to predominantly bypass such feedback: while the slight change in minus-end travel distances in the lysosomes is matched by plus-end changes (Supplementary Figure 3c, d), there is no such matching of effects on force production. Because the underlying mechanism contributing to force feedback matching opposite directions is unknown, we do not understand why such feedback fails here. However, this decoupling may be functionally useful allowing for selective directional control. In the wild-type force adaptation, minus-end forces and durations increase with attempt number, but plus-end ones stay constant. In the CDK5 KD/dominant-negative overexpression backgrounds, minus-end forces and durations decrease, but plus-end forces and durations remain unchanged (Supplementary Figure 2). Thus, at least for the cargos/cells considered here, the CDK5 pathway appears to almost entirely affect only minus-end motor activity and allows for selective directional force production control.

Finally, our findings may have implications for diabetes. LD biology is linked to insulin resistance^54^, and their transport directly affects metabolism^36^, so alteration in their trafficking by changes in force adaptation could potentially impact metabolic disease. Insulin signaling can regulate dynein to influence lysosome motility in both neurons and non-neuronal cells^55^. This involves inhibition of glycogen synthase kinase (GSK)-3β, a kinase whose dysregulation is linked to both metabolic and neurological disorders^56^. GSK-3β phosphorylation of dynein reduces its interaction with NudEL^55^. CDK5 can inhibit GSK-3β, but to our knowledge the opposite has not yet been reported^57^. Nonetheless, it will be interesting to determine whether GSK-3β modulates force adaptation, and if so, whether this occurs through altered NudEL phosphorylation by CDK5. Dynein is also required for internalization of adipocyte GLUT4 receptors in low insulin conditions^58^. Dynein force adaptation could also modulate vesicle internalization. If that is the case, alterations in force adaptation mechanisms could lead to defective glucose transport. Interestingly, KIAA0528 may be involved in GLUT4 insertion into the plasma membrane, although this is not yet completely understood^42,59^.

In summary, we found that NudEL phosphorylation control by the CDK5/14-3-3ε pathway directly regulates dynein force production in cells. It is required not only for the dynamic upregulation of dynein force underlying the previously discovered LD force adaptation but also plays a general role for other cargos such as lysosomes and mitochondria, contributing to control of their subcellular localization. Further, the differential effects of KIAA0528 (affecting LDs and mitochondria but not lysosomes) suggest cargo-specificity could in part be controlled by cargo-specific CDK5-interacting regulatory proteins, like KIAA0528. Importantly, at least for lysosomes, the CDK5 pathway is able to tune dynein’s force production capability and ultimately modulate motor tug-of-war severity; this likely seems relevant for various cellular cargos. Future work will undoubtedly explore ramifications of control of tug-of-war severity, the role and regulation of dynactin in this process, and how cargo-specific proteins like KIAA0528 mechanistically contribute to regulation.

## Methods

### Cell culture and transfections

COS-1 cells (ATCC CRL-1650) were grown in Dulbecco’s modified Eagle’s medium (Genesee) supplemented with 10% fetal bovine serum and 1% Pen–Strep at 37 °C in 5% CO_2_. Gene expression KD for CDK5, 14-3-3ε, KIAA0528, NudE, and NudEL were completed by transient transfection using commercially available siRNAs. siRNA for the control (sc-37007), CDK5 (sc-29263), 14-3-3ε (sc-29588), and KIAA0528 (sc-95830) siRNAs were obtained from Santa Cruz Biotechnology. NudE (SI00655858, SI4374356, SI4341386, and SI05147835) and NudEL (SI03246600, SI03246936, SI04264379, and SI04321191) siRNAs were obtained from Qiagen.

For the CDK5, 14-3-3ε, KIAA0528, NudE, and NudEL KDs, HiPerfect reagent (Qiagen) was used following the manufacturer’s instructions. CDK5 and KIAA0528 KDs were achieved by using a final concentration of 33 nM siRNA; 14-3-3ε KD was achieved by using a final concentration of 66 nM siRNA; and NudE and NudEL KDs were achieved by using a final concentration of 5 nM of each siRNA. (Control transfections used the same final concentration of scrambled siRNA as the target siRNA.)

For the CDK5dn overexpression and NudEL replacement, Lipofectamine-2000 (ThermoFisher Scientific) was used following the manufacturer’s instructions. Two micrograms of DNA was used for the overexpression and replacement experiments.

Transient KDs were incubated for 48 h before force measurements, WB, IP, and tracking experiments were carried out. Transient overexpression or replacement experiments were incubated for 24 h before force measurements, WB, and tracking experiments were carried out. NudEL replacement expression was induced with 20 nM doxycycline 18 h before force measurements, WB, fluorescence, and tracking experiments were carried out.

### Expression vectors

The CMV-myc-CDK5dn, pEGFP-C1-NudEL(WT), and phospho-null mutant (1-5A) expression vectors were previously described^16^.

### Mutagenesis and cloning

The NudEL-WT plasmid was mutagenized into a phospho-mimetic mutant using site-directed mutagenesis via Multi-Site-Directed Mutagenesis QuikChange Lightning (Agilent) in two rounds of mutagenesis. In the first round, F1–F3 primers were used; in the second round, P4–P5 primers were used (Supplementary Table 1). CDK5 phospho-sites on NudEL 1-5 were replaced with aspartic acid.

The three NudEL plasmids (WT, 1-5A, and 1-5D) were further mutagenized to achieve RNAi resistance. Two silent mutations were introduced in the middle of each of the four siRNA target sequences of the NudEL mRNA sequence (for a total of eight mutations/construct). The NudEL plasmids were mutagenized using site-directed mutagenesis via Multi-Site-Directed Mutagenesis QuikChange Lightning (Agilent) in two rounds of mutagenesis. F1–F3 primers (Supplementary Table 2) were used in the first round; and then F4–F6 primers (Supplementary Table 2) were used in the second round.

The three RNAi-resistant NudEL constructs were then subcloned into the expression pDONOR201 vector via Gateway Technology (Invitrogen) (Supplementary Table 3). These were then recombined into the gateway-compatible doxycycline-inducible destination vector and contained a C-terminal-S-FLAG-SBP (SFB) tag^60^.

### Immunoblotting

Cells were washed with phosphate-buffered saline (PBS) and lysed using an ice-cold 1% NP-40 buffer containing a 1× protease inhibitor cocktail (Roche) and the supernatant was collected. Proteins from the lysate were denatured using an sodium dodecyl sulfate buffer and incubating at 70 °C for 10 min. The proteins were then separated in a 4–12% Bis-tris gel (Life Technologies) and transferred to a nitrocellulose membrane through a wet transfer method. The nitrocellulose was blocked with 5% non-fat milk in Tris buffered saline (TBS: blocking buffer) for 45 min at room temperature (RT). Immunoblotting was completed with respective antibodies and blots were visualized with infrared detection on the Odyssey (Licor). Primary antibodies were diluted in blocking buffer with Tween20 at various concentrations (1:200–1:1000 v/v). Secondary antibodies were diluted in blocking buffer with Tween20 at 1:10,000 v/v. Antibodies were purchased from: Cell Signaling Technologies 2506s (CDK5) and 9635s (14-3-3ε), Novus NB110-40878 (Perilipin-2), Bethyl A301-469A-M (KIAA0528), ABClonal A5776 (NudEL), Bioss bs-5522R (pSer231NudEL), Developmental Studies Hybridoma Bank E7-s (Tubulin-β), Life Technologies (goat anti-mouse IgG 680), and Li-cor (goat anti-rabbit IgG 800). Data shown here are representative of 2–3 separate experiments.

### Force measurements in cells

Force measurements on moving LDs in COS1 cells is as described in refs. ^11,61^. LD positions in the laser trap were measured with high resolution using position sensitive detector (PSD) and cross-verified with analysis of differential interference contrast images using template matching or autocorrelation of LD intensity profile. Automated piezostage was used to position the trap at the center of linearly moving LDs toward and away from the cell center. Detachments of LDs from MT within 200 nm from the center of the laser trap were counted as escape attempts. Typically both failed and successful escape attempts could be observed in the video as LDs that failed to escape would fall back to the trap center with high velocity. However, only high-resolution PSD data (2 kHz) was used to quantify parameters of the escape attempts (position traces carried very high slope due to rapid fall back to trap center) with better accuracy. During force measurements, double trapping of LDs was quite common, and care was taken to analyze only those LDs whose motion was uninterrupted by other organelles. The ideal region of the cell for measurements is halfway between periphery and nucleus with an additional condition that there is a linear inward and outward flow of organelle traffic to rule out the ambiguity in the direction of MTs. Note that success rate of trapping linearly moving LDs that last for 5 unperturbed attempts in the cell is very low and typically we scored a maximum of 6–8 clean LD tracks in 1 h. Please note that errors in escaped fractions in each attempt (*f*) were estimated with $\sqrt {[f(1 - f)/n]}$ for *n* droplets that made the escape attempt.

### Lysosome distribution and particle tracking

After 48 h of incubation with transfection reagents, COS-1 cells were stained with NucBlue Live ReadyProbes Reagent (Life Technologies) and either 200 nM LysoTracker-Yellow or 200 nM MitoTracker-Red (Thermo Fisher Scientific) and incubated for 30 min before visualization. Live COS-1 cells were visualized at the University of California Irvine Optical Biology Core on the Zeiss LSM700 confocal laser scanning microscope. At least 30y cells were imaged. The cell perimeter, nuclear perimeter, and respective intensities were measured for each fluorescent image using ImageJ. The perinuclear region was defined as 20% of the cytoplasm surrounding the nucleus. The cell periphery was defined at the other 80% of the cytoplasm. The intensity of the cell periphery was defined as the total cell intensity minus the intensity of the perinuclear region. The percentage of total cell intensity in the cell periphery was defined as: (the intensity of the cell periphery/total cell intensity) × 100 (previously described^62^).

### LD immunofluorescence

LDs were purified from WT and siRNA-treated COS-1 cells using a sucrose gradient as previously described^11^. Probing for the presence of proteins on the surface of LDs is carried out as described below. A sample chamber constructed with 0.17 × 40 × 22 mm^3^ clean coverslips, double-sided adhesive tape, and microscope cover glass to hold about 20 μl of solution. Coverslips were cleaned with KOH solution followed by ddH_2_O and ethanol in ultrasonic bath. About 25 μl of as-purified LDs in buffer were flown into the chamber and incubated for 10 min at RT. LD surface was blocked with 10% bovine serum albumin (BSA) in PBS for 20 min, followed by incubation with primary antibodies to a final concentration of 2 μg/ml in PBS with 10% BSA and 0.1% Triton X. Unattached primary antibody (1:200 diluted) was washed by flushing the chamber with 30 μl of PBS with 10% BSA twice before adding secondary antibody. Primary antibodies used were: CDK5 (Cell Signaling 2506 S), KIAA0528 (Bethyl A301-468A), 14-3-3ε (Invitrogen 5H10L5), and green fluorescent protein (GFP; Cell Signaling 2912 S). Fluorescence imaging was carried out after 1-h incubation with secondary antibody (0.02 mg/ml, Alexa Fluor 488-labeled anti-Rabbit) in the imaging buffer (80 mM PIPES, 1 mM MgCl_2_, 2 mM EGTA, 1 mg/ml casein, and 1 mM dithiothreitol). An EMCCD camera (quantEM 512, Photometrics) and a single-mode, 488 nm Ti-Sapphire excitation laser in semi-TIRF configuration were used. Fluorescence signal integration time was fixed at 0.5 s.

### Eccentricity and run distances of lysosomes

Cells were incubated with 200 nM of Lysotracker red DND-99 (Life Technologies) for 30 min in the medium used for tissue culture. A sample chamber similar to the one adopted for force measurements was used to image the cells. Images were acquired in semi-TIRF mode at 10 fps using 568 nm excitation laser and the quantEM 512 camera. Spatial resolution of the acquired images in the above set-up is 60 nm/pixel.

Eccentricity of lysosomes in each movie frame was determined using custom Matlab code for image processing. One hundred consecutive images for each cell (18 cells in each condition from two experimental replicates) were used for this analysis, because the deformations could be dynamic, and we wanted to catch them if they occurred; the distributions shown thus reflect instantaneous multiple determinations of each lysosome’s eccentricity. If a specific lysosome was visible in all 100 consecutive frames, and it were circular in the first 50 frames, and very stretched in the next 50, it would have yielded 50 eccentricity measurements that were 0, and 50 that were 1, all included in the histogram. Binary images were generated from the raw images, and the eccentricity and areas of individual lysosomes were measured (after appropriate filtering to reduce the background). Eccentricity values range from 0 to 1, with 0 corresponding to a perfect circle and 1 representing highly deformed objects. Only lysosomes with areas in the range of 8–200 square pixels were considered.

Particle tracking was carried out by custom softwares LVcorr and Marathon. LVcorr was used to generate long tracks (track length ~100 s and 90 tracks from 18 cells in each condition) from the videos recorded above. Using tracks files from LVcorr as input, the parsing software Marathon was used to extract the pauses and run distances of lysosomes moving linearly in kinesin and dynein directions.

### Immunoprecipitation

Cells were lysed and NudEL was immunoprecipitated using the Pierce Crosslink Magnetic IP/co-IP Kit (Thermo Scientific 88805) following the manufacturer’s instructions. Ten micrograms of NudEL antibody (abcam ab25959) was used for each IP. Elutions were then run on a WB probing for NudEL and pSer231NudEL.

### Statistics

All graphs are mean and s.e.m. Statistical significance was determined by Student’s *t* test, test of proportions, Kolmogorov–Smirnov test, and Wilcoxon sign-ranked *t* test. *P < 0.05, **P < 0.01, ***P < 0.001, ****P < 0.0001.

### Code availability

LVcorr and Marathon are freely available on GrossLab website. Lysosomal eccentricity estimation code is available upon request by email to corresponding author.

## Supplementary information

Supplementary Information

Supplementary Movie 1

Supplementary Movie 2

Description of Additional Supplementary Files

Supplementary Info File - reporting summary

## Data Availability

Data supporting the findings of this manuscript are available from the corresponding author upon reasonable request.
