# Supplementary material for: Regulation of in vivo dynein force production by CDK5 and 14-3-3ε and KIAA0528
Source: Nat Commun. 2019 Jan 16;10:228. doi: 10.1038/s41467-018-08110-z (PMC6335402; doi:10.1038/s41467-018-08110-z)
Supplement: Supplementary file 1 — Supplementary Information [file 41467_2018_8110_MOESM1_ESM.pdf]

Supplementary Information for

Regulation of in vivo dynein force production by CDK5 and 14-3-3ε and KIAA0528

Chapman *et al*

Supplementary Figures

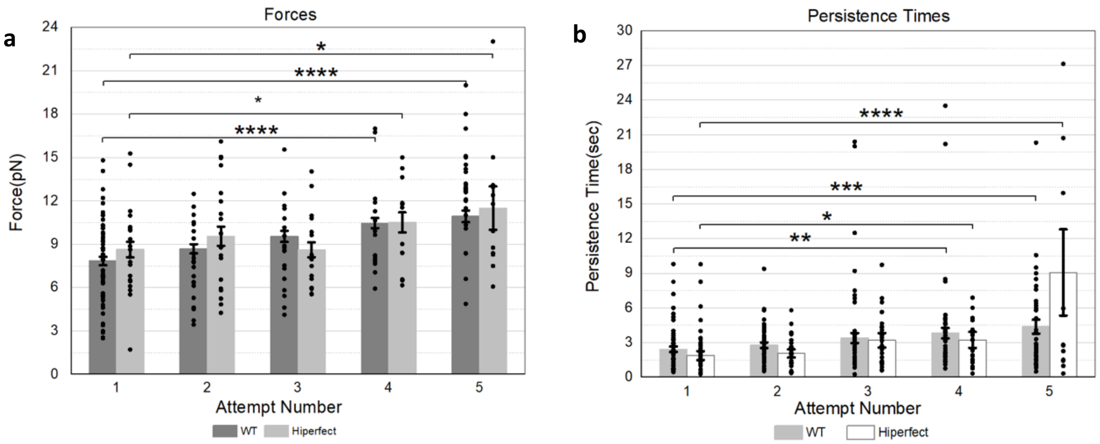

Supplementary Figure 1. Transfection reagent (control) does not affect dynein force adaptation

*Average minus-end peak forces (**a**) and persistence times (**b**) were similar between wildtype cells (darker grey in each panel) and wildtype cells treated with HiPerfect (lighter grey in each panel). Quantified data represent the mean $\pm$s.e.m. of $n \geq 5$ independent experiments. T-test p values * P <0.05, ** P <0.01, ***P <0.001, ****P < 0.0001*

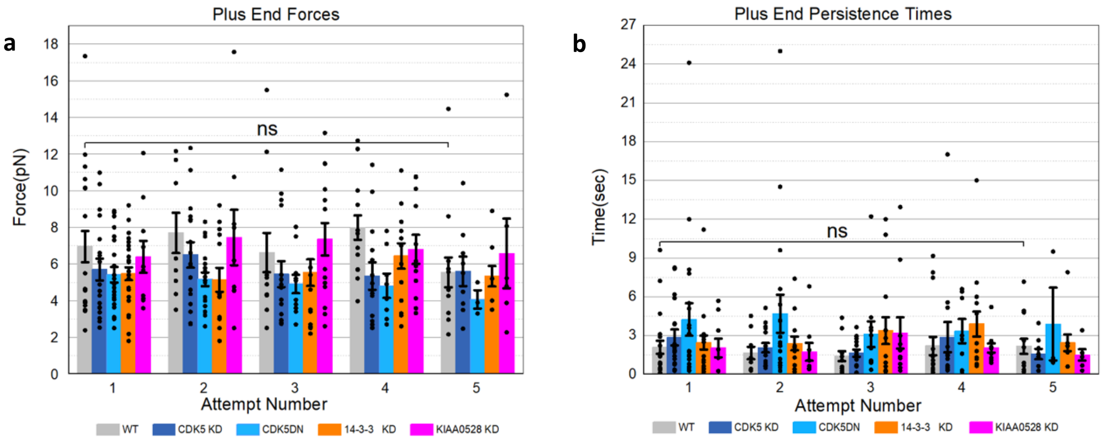

Supplementary Figure 2. Plus-end forces and persistence times show no force adaptation under any conditions

*Average peak forces (**a**) and persistence times (**b**) from plus-end moving LD cargo do not increase at each attempt in WT, CDK5 knockdown, CDK5dn overexpression, 14-3-3ε knockdown, and KIAA0528 knockdown backgrounds. Quantified data represent the mean $\pm$s.e.m. of $n \geq 4$ independent experiments. Force and persistence times values at each attempt were compared via ANOVA within each sample. All t-test p-values > 0.05*

a

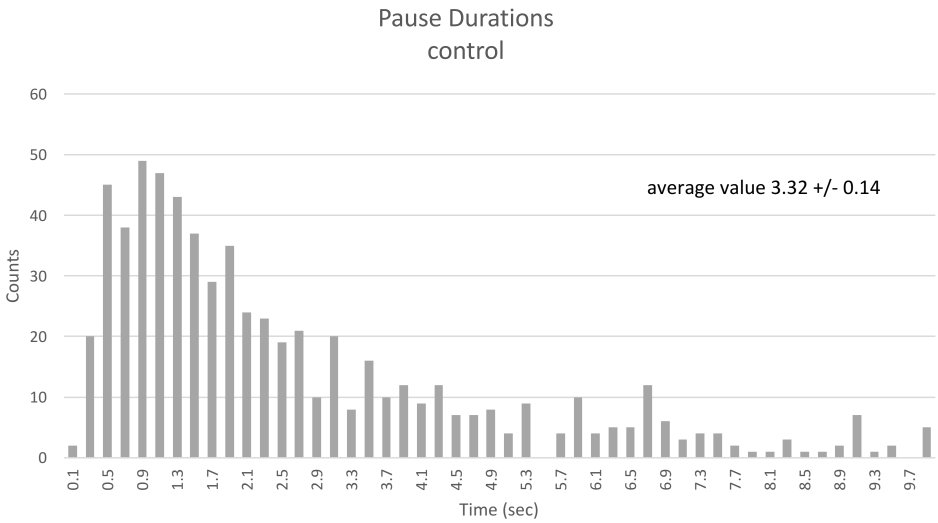

Pause Durations
control
average value 3.32 +/- 0.14
Counts
Time (sec)

b

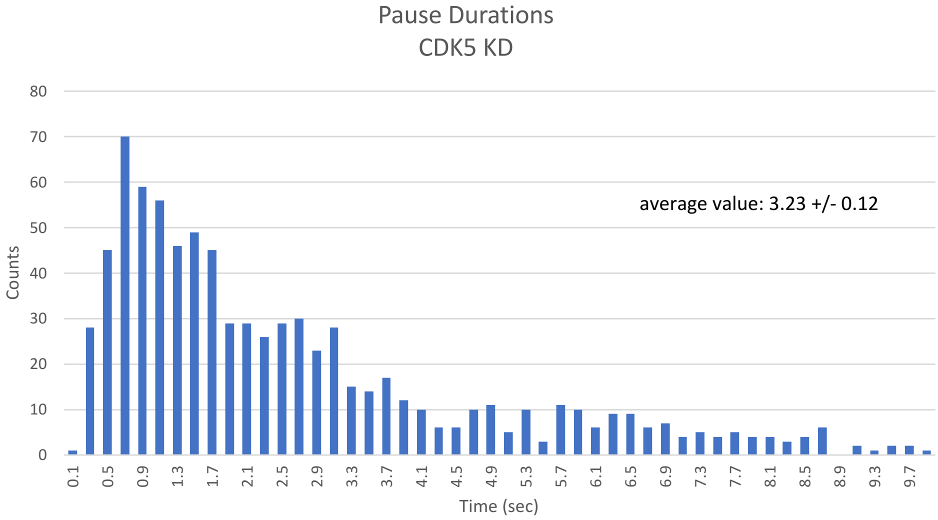

Pause Durations
CDK5 KD
average value: 3.23 +/- 0.12
Counts
Time (sec)

c

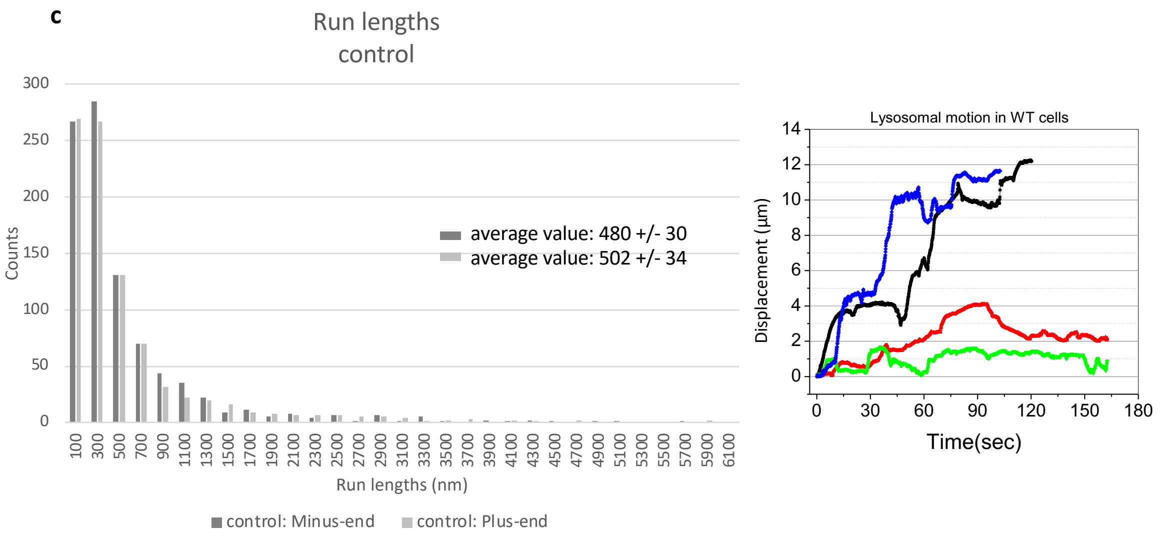
Run lengths
control
Counts
average value: 480 +/- 30
average value: 502 +/- 34
Run lengths (nm)
control: Minus-end
control: Plus-end
Lysosomal motion in WT cells
Displacement (μm)
Time(sec)

d

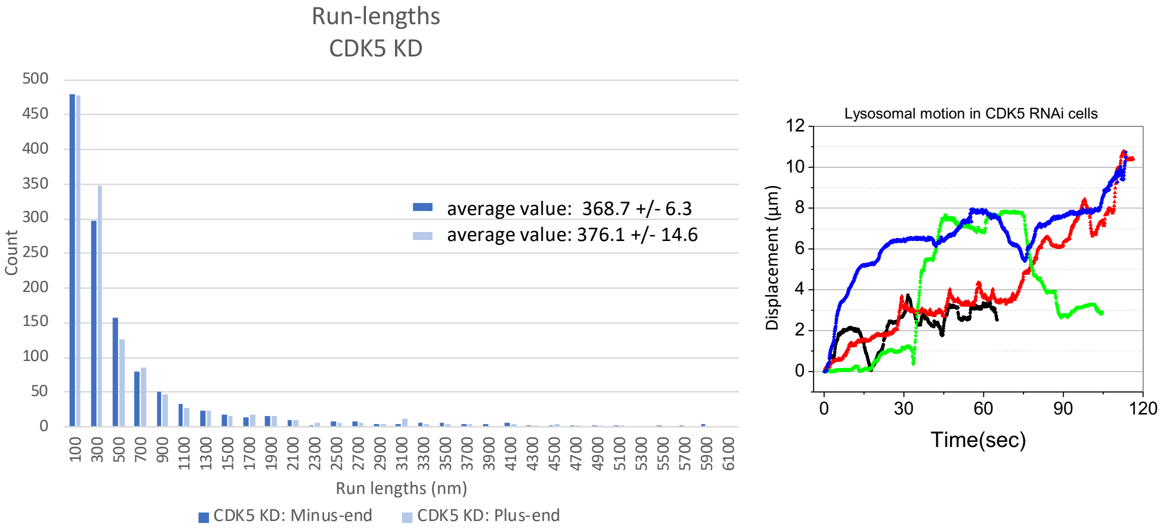
Run-lengths
CDK5 KD
Count
average value: 368.7 +/- 6.3
average value: 376.1 +/- 14.6
Run lengths (nm)
CDK5 KD: Minus-end
CDK5 KD: Plus-end
Lysosomal motion in CDK5 RNAi cells
Displacement (μm)
Time(sec)

e

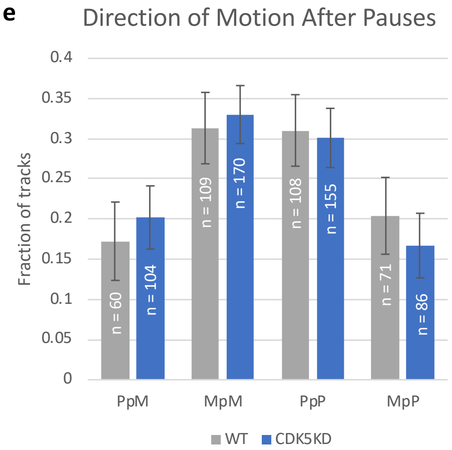
Direction of Motion After Pauses
Fraction of tracks
n = 60
n = 104
n = 109
n = 170
n = 108
n = 155
n = 71
n = 86
PpM
MpM
PpP
MpP
WT
CDK5KD

Supplementary Figure 3. Most aspects of lysosomal motion are not altered by changes in Tug-of-war severity
*Particle tracking analysis of lysosomes shows no pause duration profile differences between control cells (**a**) and CDK5 knockdown cells (**b**), a small run length difference between control (four samples traces are included) (**c**) and CDK5 knockdown (four samples traces are included) (**d**) but no effect on the relative difference between plus-end run lengths and minus-end run lengths, and no significant differences in directional changes after pausing (**e**). PpM: lysosome moving towards the plus-end of MT, pauses, then moves towards the minus-end of MT,*
MpM: lysosome moving towards the minus-end of MT, pauses, then moves towards the minus-end of MT,
PpP: lysosome moving towards the plus-end of MT, pauses, then moves towards the plus-end of MT,
MpP: lysosome moving towards the minus-end of MT, pauses, then moves towards the plus-end of MT
Quantified data represent fraction of tracks ± standard error of proportions. n = 2 independent experiments for a-d; n = 1 for e

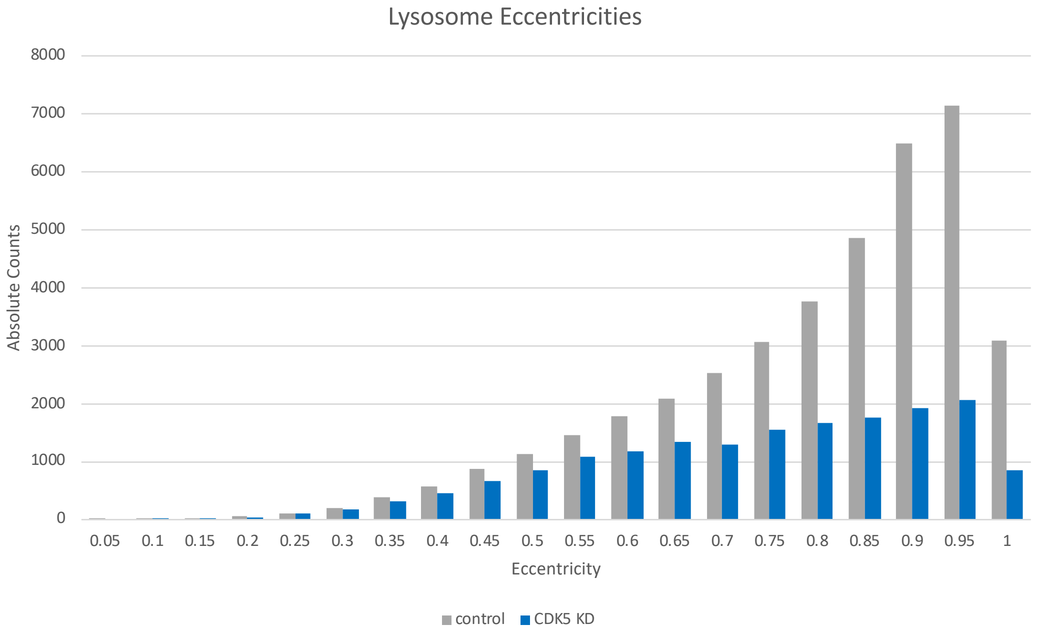

Supplementary Figure 4. Absolute counts of lysosome eccentricities from Figure 8 also show a difference in distribution
(Wilcoxon Sign-Ranked t-test p value 0.00014)

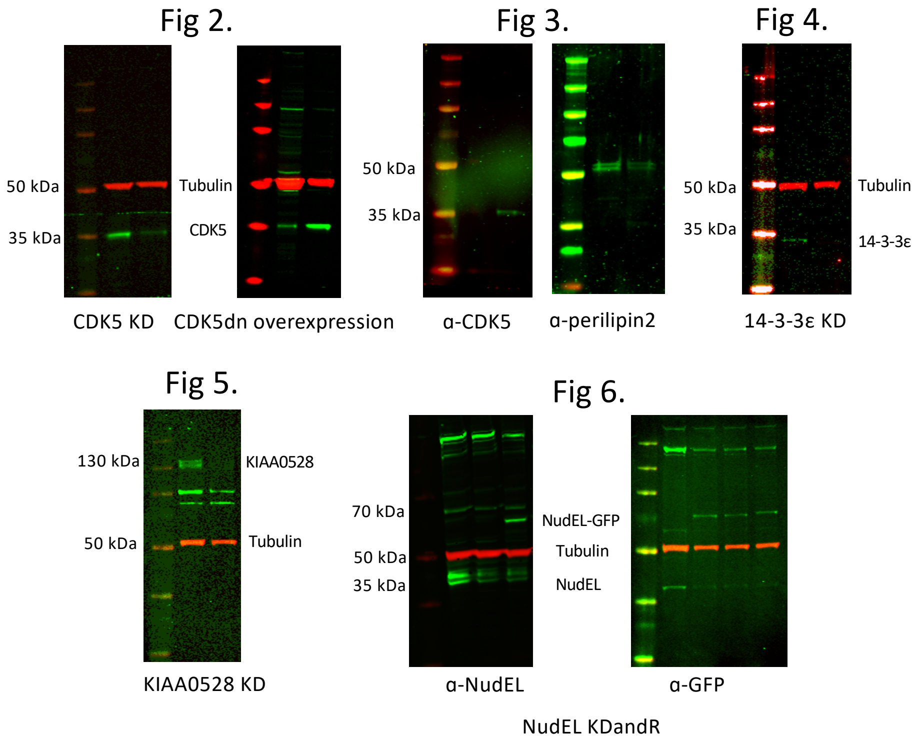

Supplementary Figure 5. Full WBs presented in article

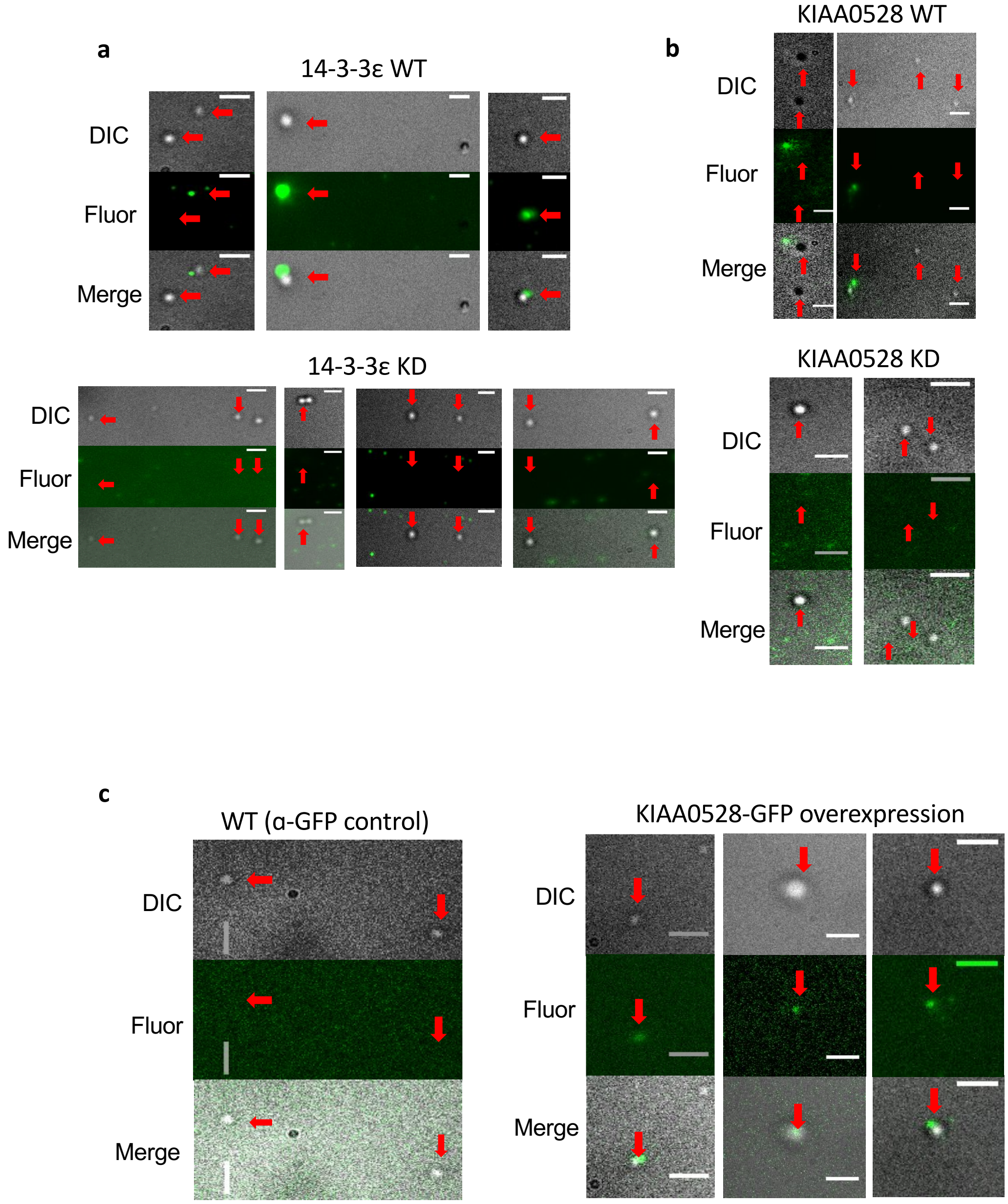

Supplementary Figure 6. representative LDs from immunofluorescence (Figure 3)

*Top four panels are sample images from immunofluorescence experiments conducted with antibodies against 14-3-3ε (**a**) and KIAA0528 (**b**). The two bottom panels are from immunofluorescence experiments conducted with anti-GFP antibody (**c**). LDs labeled with red arrows, 2μm scale bar and red arrows to designate lipid droplets.*

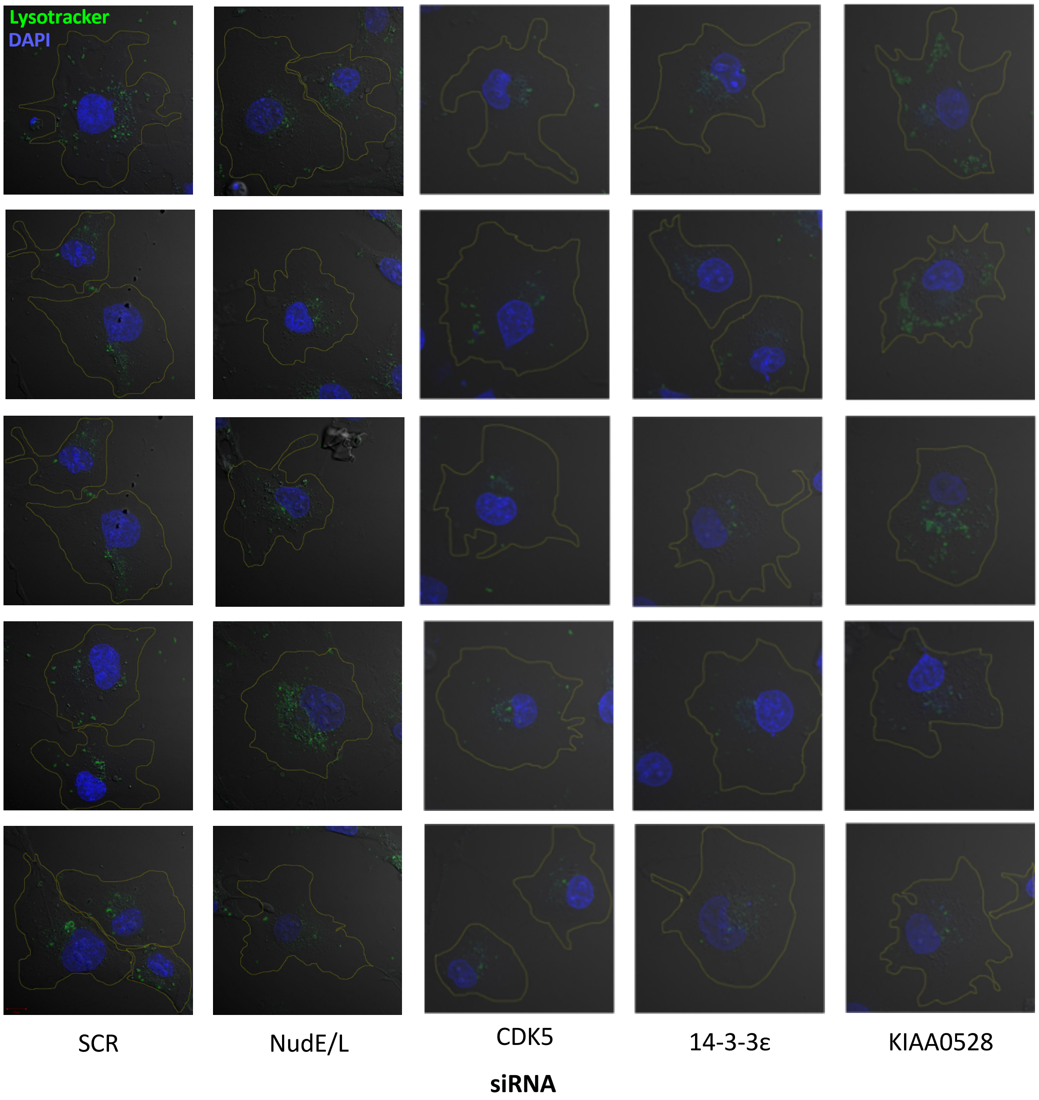
Lysotracker
DAPI
SCR
NudE/L
CDK5
14-3-3ε
KIAA0528
siRNA

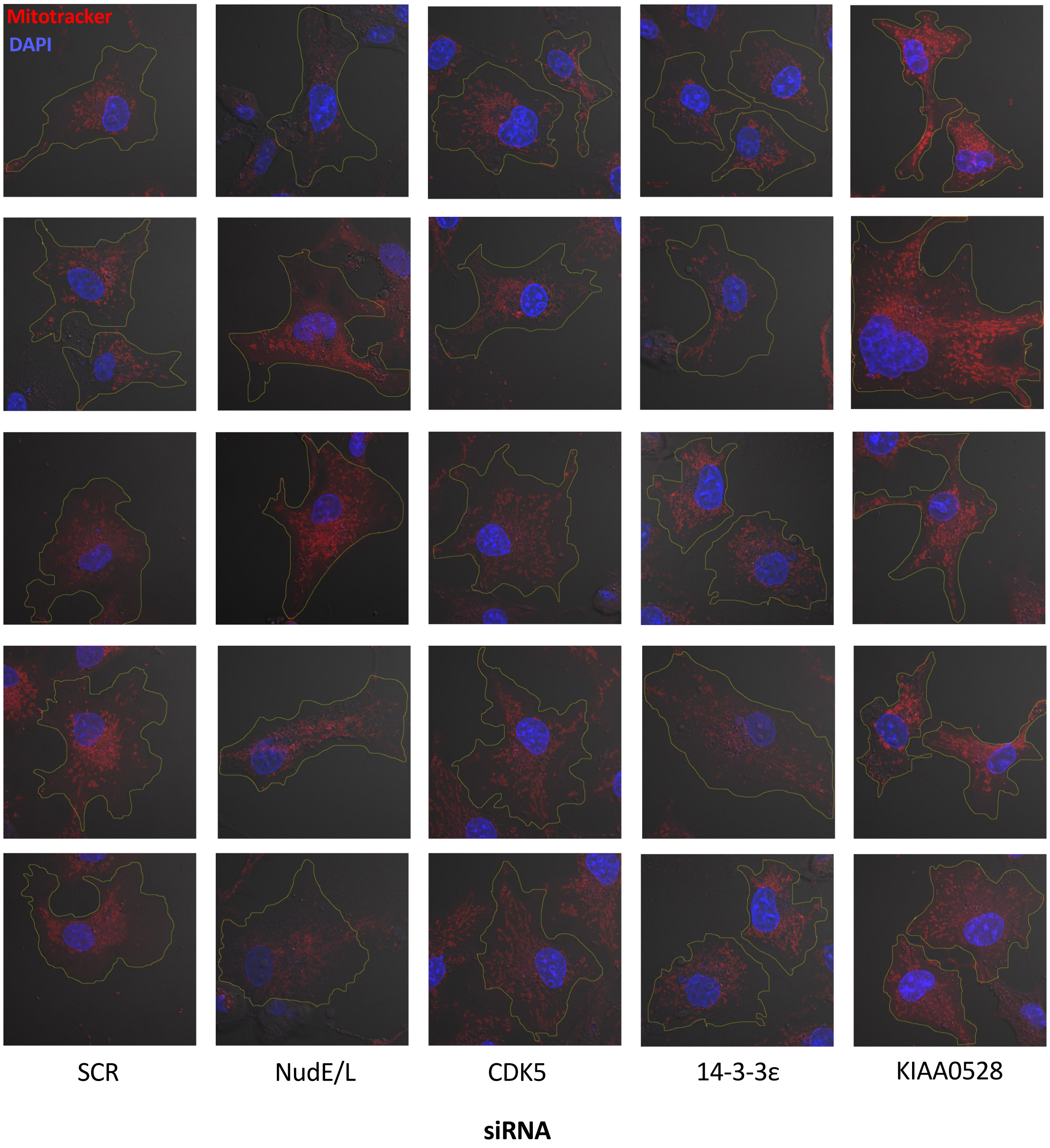

Supplementary Figure 7. representative images from Lysotracker and Mitotracker experiments (Figure 7)
Lysosomes are labeled by Lysotracker in green, mitochondria are labeled by Mitotracker in red, nuclei are labeled by DAPI in blue. Five images are shown for each condition.

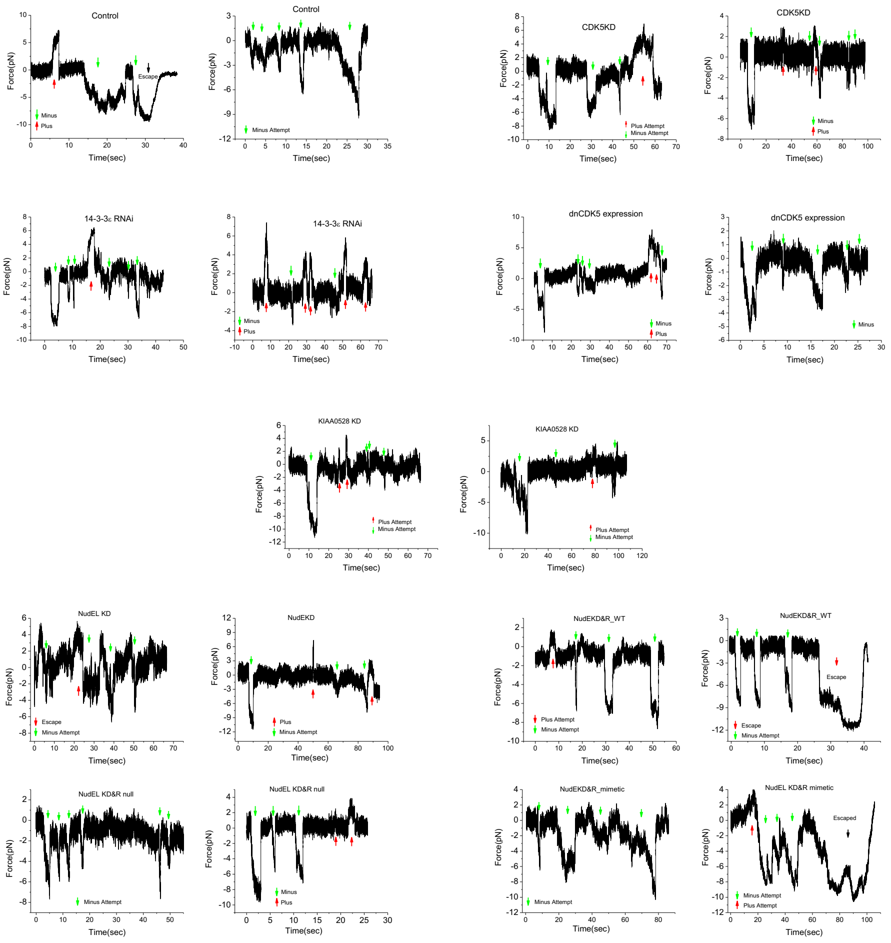

Supplementary Figure 8. Sample traces of force measurement experiments

Supplementary Table 1 Oligos used in phospho-mutagenesis

Oligo Name	DNA Sequence 5'-3'
GFP-NudEL_S197D_F1	GCA GAG GGA GTT AGT GGA TCG GTA CCA AAA CCA TTT GG
GFP-NudEL_T219D_F2	CAG TCT AGA GTT GGA GAG TCA GGA GCC GAC TTT CTA
GFP-NudEL_S242D_F3	GTT CCT TTG CCA ACA GGG TCA GCT GGC AAA GAA AGT GAT GC
GFP_NudEL1-5D_mutagen2_P4	CCT AGC AGA GGG ATC TAG TGG ATC GGT ACC
GFP_NudEL1-5D_mutagen2_P5	GGT ATA GCT TCC GGA TCA GGA AAA GTG TTC TCC GTT CC

Supplementary Table 2 Oligos used in RNAi-resistant mutagenesis

Oligo Name	DNA Sequence 5'-3'
NudEL-RNAiResistant_F1	GGA GAA AGA GCA CCA ATA TGC ACA GAG C
NudEL-RNAiResistant_F2	CTC AGT GTT AGA GGA TGA TTT AAG TCA GAC TCG CGC CAT TAA GG
NudEL-RNAiResistant_F3	CAG TGG AAG TTG AGC AAA GGC TAA ACC
NudEL-RNAiResistant_F4	GGA GAA GCT AGA ACA CCA ATA TGC ACA GAG C
NudEL-RNAiResistant_F5	CTC AGT GTT AGA GGA CGA TTT AAG TCA GAC ACG CGC CAT TAA GG
NudEL-RNAiResistant_F6	CAC TGG AAG ACT TTG AGC AGA GGC TAA ACC

Supplementary Table 3 Oligos used to insert NudEL into pDONOR201

Oligo Name	DNA Sequence 5'-3'
GFP-NudEL_RNAiR4_F	GGG GAC AAG TTT GTA CAA AAA AGC AGG CTC CAT GGT GAG CAA GGG CGA GGA GCT GTT C
GFP-NudEL_RNAiR4_BP_Rc	GGG GAC CAC TTT GTA CAA GAA AGC TGG GTT TCA CAC ACT GAG AGG CAG CAT ACC CGG
