# Supplementary material for: Regulation of in vivo dynein force production by CDK5 and 14-3-3ε and KIAA0528
Source: Nat Commun. 2019 Jan 16;10:228. doi: 10.1038/s41467-018-08110-z (PMC6335402; doi:10.1038/s41467-018-08110-z)
Supplement: Supplementary file 4 — Description of Additional Supplementary Files [file 41467_2018_8110_MOESM4_ESM.docx]

**Tile:** Supplementary Movie 1
**Description:** Sample lysosome particle tracking video data from control cells

**Tile:** Supplementary Movie 2
**Description:** CDK5 knockdown cells
